# Solvents of Iodoform

**Published:** 1879-08

**Authors:** 


### SOLVENTS OF IODOFORM.

Dr. Vulpius, of Heidelberg, points out that the usual statements regarding the solubility of Iodoform in alcohol and ether are incorrect. He speaks of collodion as one of the most useful solvents—one part of Iodoform, previously shaken up with a little ether is readily soluble in nine parts of collodion. This will be found a convenient method of applying it in many cases.

